# Venous Thromboembolism Prophylaxis in Inflammatory Bowel Disease: A Two-year Retrospective Study of Patients Presenting With Inflammatory Bowel Disease to a Community Hospital

**DOI:** 10.7759/cureus.29178

**Published:** 2022-09-15

**Authors:** Dominic Amakye, Onoriode Kesiena, Ademayowa Ademiluyi, Margaret Gavor, Zahraa Rabeeah

**Affiliations:** 1 Graduate Medical Education, Piedmont Athens Regional Medical Center, Athens, USA; 2 Internal Medicine, Piedmont Athens Regional Medical Center, Athens, USA

**Keywords:** prophylactic anticoagulation, anticoagulation, venous thromboembolism (vte), venous thromboembolism prophylaxis, flare, inflammatory bowel disease

## Abstract

Objective

We set out to determine the rate of pharmacological venous thromboembolism (VTE) prophylaxis among patients admitted with inflammatory bowel disease (IBD) and indirectly compare it to national trends. We also assessed the demographic and clinical correlates for non-prescription of pharmacologic VTE prophylaxis among IBD patients with and without a flare.

Methods

We extracted data from 123 patients admitted to our facility with IBD from September 2018 to August 2020 retrospectively. Out of this cohort, 26 patients were excluded and 96 were included in our analysis. Baseline characteristics were analyzed using descriptive statistics. Multiple logistic regression was used to evaluate the correlates of pharmacological VTE prophylaxis use in individuals with IBD and to analyze the predictors of VTE prophylaxis use in patients with IBD flares.

Results

Out of the 96 patients with IBD included in this study, 61 (63.5%) presented with an IBD flare, and among those with a flare, 26/61 (42.6%) received VTE prophylaxis. IBD patients aged ≥ 65 years and of Black race were less likely to be placed on pharmacological VTE prophylaxis (adjusted odds ratio (AOR) 0.20, 95% CI (0.06 - 0.70), p-value 0.012) and (AOR 0.16, 95% CI (0.05 - 0.50), p-value 0.002) respectively. Among those with a flare, the presence of bright red bleeding per rectum was associated with a low rate of pharmacologic VTE use (AOR 0.01, 95% CI (0.00 -1.78), p-value 0.001). Overall the rate of VTE prophylaxis use in the IBD patient cohort was 56.3% and this was irrespective of flare status.

Conclusion

Our study showed the low rate of pharmacologic VTE prophylaxis use in IBD patients at this center and this finding was in line with national trends. Interestingly age and the race of patients played a major role in the decision to provide pharmacological VTE prophylaxis but the reason for this finding was not explored by our study. A larger multi-center study is needed to further evaluate these relationships.

## Introduction

Inflammatory bowel disease (IBD) is a chronic remitting, progressive inflammatory condition that involves the gastrointestinal tract with associated extra-intestinal manifestations [1]. IBD is made up of two distinct disease entities; Crohn’s disease (CD) and ulcerative colitis (UC) [2]. IBD is thought to largely occur from an interplay between environmental, genetic, and immune factors but the exact etiology is unknown [3]. The prevalence of IBD is increasing globally and in the US, 1.3% of the adult population are reported to have IBD [4]. Patients with IBD often have extensive morbidities, often requiring multiple hospitalizations with increased healthcare costs [5].

One of the well-recognized extraintestinal manifestations of IBD is venous thromboembolism (VTE) [6]. The risk of VTE is about two to three times higher in patients with IBD compared to the general population and this risk increases by about eightfold during an acute flare of IBD [6-8]. The pathogenesis of VTE in IBD is multifaceted and not fully understood. The up-regulation of acute phase reactants and coagulation factors from chronic systemic inflammation are some of the suggested drivers of clot formation [7,8]​​​​​​. In acute IBD flares, the active systemic inflammation combined with immobilization during hospitalization both contribute to the development of VTE [9].

Despite the increased VTE risk in IBD, the use of pharmacologic VTE prophylaxis is reported to be low in this group of patients [8]. Moreover, in cases where an acute flare is associated with GI bleeding, VTE prophylaxis is mostly withheld [10], mainly due to safety concerns [6]. However, multiple studies have shown pharmacological VTE prophylaxis to be safe among patients with IBD flare presenting with rectal bleeding [11-13]. Current guidelines from the American Thoracic Society and the Canadian Gastroenterology Association recommend pharmacologic VTE prophylaxis for IBD patients admitted to the hospital because of the increased risk of clot formation [9].

In this study, we assess the use of pharmacologic VTE prophylaxis in IBD patients admitted to a community hospital.

## Materials and methods

Study design

This is a single-center retrospective study of patients admitted with a diagnosis of inflammatory bowel disease with or without a flare. The data were abstracted from a retrospective chart review of electronic health records. IRB approval, number 1774973-1, was obtained from the Piedmont Athens Regional Institutional Review Board.

Study population

A total of 123 patients admitted between September 2018 to August 2020 were identified using ICD-10 codes “ulcerative colitis - K50.90”, “Crohn’s disease - K51.90”, and “IBD - K52.9”. This included individuals with a previous diagnosis and those that were newly diagnosed. Adults ≥ 18 years who were admitted with these ICD-10 codes were included in this study, including those with an acute flare. The case definition of an acute flare of UC was the passage of more than six bowel movements in 24 hours with or without systemic signs of toxicity. For CD, an acute flare was based on the patient's subjective symptoms. A total of 27 patients were excluded and these included; anticoagulation for VTE prior to admission, chronic anticoagulation for atrial fibrillation and valvular heart disease, patients with bleeding disorders who presented with active bleeding, and those with massive gastrointestinal bleed (GIB) with associated hemodynamic instability. Our institution has a VTE prophylaxis policy and this recommends intermittent pneumatic compressions and or pharmacological VTE prophylaxis for every admitted patient with a moderate VTE risk. Moderate VTE risk is at least one risk factor for VTE including obesity, and immobility. However, utilization is at the provider's discretion. 

Patient characteristics 

The demographic characteristics of interest were age, sex, ethnicity, smoking status, and body mass index (BMI). Age was classified as < 40, 40 - 64, and >64 years. Gender as male and female, ethnicity as Caucasians and Blacks/African Americans, and smoking status as smokers and non-smokers.

The clinical characteristics of interest were hemoglobin level on admission, C-reactive protein (CRP), chronic steroid use, type of IBD, and flare status. Hemoglobin status on admission was classified as <12.0 g/dL and ≥12.0 g/dL, CRP as <3 g/dL and ≥3 g/dL, and chronic steroid use as “on chronic steroid” and “not on chronic steroid”. IBD type was also classified as UC and CD, and IBD flare status as the presence of a flare and absence of a flare. 

Outcome variable

The outcome variable was the use of either unfractionated heparin (UFH) or low molecular weight heparin (LMWH) for VTE prophylaxis while on admission. 

Statistical analysis

We described the background characteristics of the study population using descriptive statistics. Continuous variables were reported as means (standard deviation) and medians (interquartile range), while categorical variables were reported as frequencies (percentages). The correlates of VTE prophylaxis use in individuals with IBD and predictors of VTE prophylaxis use in patients with IBD flares were analyzed using multiple logistic regression models independently. Co-variates were adjusted for in each regression model. Data were analyzed using Stata Statistical Software v.14 (StataCorp LP, College Station, TX) with a set p-value of < 0.05.

## Results

Table 1 shows the baseline characteristics of the study population. The median (IQ) age was 40 (32.5) years. Around 51% of the study participants were females, 70.8% were Caucasians and 66 (68.8%) were non-smokers. CD was the major IBD type with 71.9% of the study population having this. About 56.3% of those with IBD received pharmacological VTE prophylaxis. Among those with a flare, 42.6% received VTE prophylaxis. The median (IQ) BMI was 24.8 (12) kg/m2 and the mean (SD) hemoglobin (g/dL) was 11.5 (2.6) g/dL.

**Table 1:** Baseline characteristics of the population VTE: venous thromboembolism

Characteristics	Median (IQ), means (SD), Frequency (%)
Age, years (median, IQ)	40 (32.5)
Sex	
Male	47 (49.0%)
Female	49 (51.0%)
Ethnicity	
Blacks/African Americans	28 (29.2%)
Caucasians	68 (70.8%)
Smoking status	
Non-smokers	66 (68.8%)
Smokers	30 (31.2%)
Body mass index (median, IQ)	24.8 (12)
Mean (SD) hemoglobin on admission, g/dl	11.5 (2.6)
Type of inflammatory bowel disease	
Crohn’s disease	69 (71.9%)
Ulcerative colitis	27 (28.1%)
Inflammatory bowel disease flare	
No flare	35 (36.5%)
Flare	61 (63.5%)
Chronic steroid use	
Not on chronic steroid	66 (68.8%)
On chronic steroid	30 (31.3%)
VTE prophylaxis use	
No VTE prophylaxis	42 (43.7%)
Received VTE prophylaxis	54 (56.3%)

Table 2 shows the sociodemographic and clinical correlates of VTE prophylaxis use in individuals with IBD regardless of acute flare status. Patients with IBD regardless of flare status aged ≥ 65 years old had less than 65% odds of receiving pharmacological VTE prophylaxis (OR 0.35, 95% CI (0.13 - 0.99), p-value 0.047). This relationship remained significant with a further reduction of the odds to 80% after adjusting for covariates (adjusted odds ratio (AOR) 0.20, 95% CI (0.06 - 0.70), p-value 0.012). Black ethnicity correlated with less than 82% odds of receiving pharmacological VTE prophylaxis in patients with IBD regardless of flare status (OR 0.18, 95% CI (0.06 - 0.54), p-value 0.002). This relationship persisted and remained about the same after adjusting for covariates (AOR 0.16, 95% CI (0.05 - 0.50), p-value 0.002).

**Table 2:** Sociodemographic and clinical correlates associated with pharmacological venous thromboembolic prophylaxis use in individuals with inflammatory bowel disease ** controlled for smoking status and parts of bowel involved AOR: adjusted odds ratio

Factors	OR (95% CI)	p-value	AOR (95% CI)**	p-value
Age category				
<40	1		1	
40 to 64	1.91 (0.67 – 5.44)	0.226	2.22 (0.71 – 6.93)	0.171
>65	0.35 (0.13 – 0.99)	0.047	0.20 (0.06 – 0.70)	0.012
Gender				
Female	1		1	
Male	1.55 (0.69 – 3.48)	0.293	1.38 (0.58 – 3.25)	0.468
Ethnicity				
Caucasians	1		1	
Black/African American	0.18 (0.06 – 0.54)	0.002	0.16 (0.05 – 0.50)	0.002
Hemoglobin				
<12 g/dL	1		1	
>12 g/dL	1.63 (0.72 – 3.69)	0.246	2.04 (0.84 – 4.99)	0.118
C-reactive protein levels				
<3 g/dL	1		1	
>3 g/dL	0.44 (0.13 -1.44)	0.173	0.51 (0.15 – 1.76)	0.286
Chronic steroid use				
Not on steroids	1		1	
On steroids	0.47 (0.19 -1.12)	0.088	0.57 (0.22 -1.47)	0.245

Table 3 outlines the predictors of pharmacological VTE prophylaxis use in individuals with an IBD flare. Hematochezia was the only significant negative predictor of pharmacological VTE use in patients with an IBD flare (AOR 0.01, 95% CI (0.01 - 0.18), p-value 0.001). Specifically, patients with an IBD flare who present with bright red bleeding per rectum have less than 99% odds of being placed on pharmacological VTE prophylaxis as compared to those with an acute flare who do not present with hematochezia.

**Table 3:** Predictors of pharmacological venous thromboembolism prophylaxis use in patients with an acute inflammatory bowel disease flare IBD; inflammatory bowel disease; UC: ulcerative colitis; BMI: body mass index

Predictors	OR (95% CI)	p-value
Gender (male)	0.52 (0.08 – 3.25)	0.485
IBD (UC)	1.57 (0.27 - 9.26)	0.621
Ethnicity (Caucasian)	0.19 (0.02 – 1.58)	0.125
Smokers	0.43 (0.10 - 1.89)	0.265
Hemoglobin on admission	0.98 (0.72 - 1.32)	0.882
Age	1.01 (0.95 - 1.06)	0.779
Hematochezia	0.01 (0.01 - 0.18)	0.001
BMI	1.05 (0.94 - 1.18)	0.36
CRP	0.80 (0.58 - 1.09)	0.16

## Discussion

We set out to determine the proportion of patients admitted with IBD who were prescribed pharmacological VTE prophylaxis, and then evaluate the sociodemographic and clinical correlates of VTE prophylaxis use in this group of patients. We also analyzed predictors of VTE prophylaxis use in those with IBD flares and compared these with national trends. We found out that the majority of IBD patients presenting to our facility had CD (71.9%). The rate of VTE prophylaxis use was 56% among patients admitted with IBD and 42.6% among those with an IBD flare. Patient demographic factors such as age > 65 years and Black race were associated with low odds of receiving VTE prophylaxis. Similarly, patients presenting with an acute flare associated with bright red bleeding per rectum also had lower odds of receiving VTE prophylaxis.

The challenge regarding VTE prevention in hospitalized IBD patients is the low implementation of pharmacological VTE prophylaxis [8]. The VTE prophylaxis rate of 56.3% in our study is lower than rates reported in North America. In a study of 974 patients with IBD admitted to Mount Sinai Hospital, Toronto, Canada from 2010 to 2012, the overall pharmacological prophylaxis rate was 68%^ ^[11]. Tinsley et al. also reported a pharmacological prophylaxis rate of 67.6%, in 377 patients with UC admitted to Mount Sinai Medical Center, New York City from 2007 to 2011 [6]. Similar studies at the Beth Israel Deaconess Medical Center, Boston, Massachusetts, and at Truman Medical Center, Kansas City, Missouri, however, reported a rate of VTE prophylaxis use of 37% and 39.7% respectively [12,13]. The lower rate of VTE prophylaxis use in these latter studies is likely due to the study population consisting solely of patients with IBD flares compared to our study where 63.5% of the patients had flare-ups.

In our patient cohort, we found out that older age and Black race were associated with reduced odds of receiving pharmacologic VTE prophylaxis irrespective of their IBD flare status. These associations are new and were not reported in earlier studies. The lower likelihood of these sub-group patients receiving pharmacological VTE prophylaxis, despite reports of higher VTE risk is worrying considering they are also at risk of disparities in their care [14-21]. Furthermore, given a significant part of our patient population, 30% were blacks and 25% were aged 65yrs and above, this likely contributed to the lower pharmacological VTE prophylaxis rate.

For patients who presented with a flare, the presence of hematochezia (bright red bleeding per rectum) was associated with a low likelihood of receiving VTE prophylaxis and this finding was consistent with reports from other studies [10,11]. The commonly reported reason for holding VTE prophylaxis is safety concerns on the part of the provider [6]. However, IBD patients presenting with a flare tend to have a high incidence of VTE and also high mortality when they do develop VTE [6-8]. Pharmacologic VTE prophylaxis is reported to be largely safe except in instances where there is hemodynamic instability [9].

The initial hemoglobin on admission did not have a predictive effect on VTE prophylaxis, which was consistent with the report from Faye et al [10]. Patient characteristics such as gender and smoking status had no significant impact on VTE prophylaxis use in our study population. Interestingly, these same factors were associated with higher odds of VTE prophylaxis use in another study by Kaddourah et al [12]. The majority of patients in our study were non-smokers (66%) compared to 50.2% in the study by Kaddourah et al. [12] and this may have played a role in our unique finding.

A notable strength of our study is the indirect comparison of the pharmacological VTE prophylaxis rates with national trends, which puts into perspective how well physicians are adhering to evidence-based guidelines on the care of IBD patients. Additionally, unlike most studies that evaluated only pharmacological VTE prophylaxis in IBD patients with an acute flare, this study also evaluated this intervention among patients without flares. The incorporation of this cohort in our analysis was important because patients with IBD even without an acute flare are still at increased risk of VTE compared to the general population [6-8].

The study has some limitations. It is a retrospective single-center study and the extrapolation of findings to other facilities may be inappropriate. Secondly, the smaller number of patients involved in the study may have reduced the power of the study hence the findings and associations in this study may be different in larger studies. To resolve these limitations, larger multicenter studies need to be conducted in the future. 

## Conclusions

In conclusion, the rate of pharmacologic VTE prophylaxis use is lower in our hospital as compared to national rates. Patients of Black/African American ethnicity and older patients with IBD are less likely to receive pharmacological VTE prophylaxis on hospital admission irrespective of their flare status. Also, patients with IBD flares are less likely to receive pharmacological VTE prophylaxis if they present with hematochezia. These findings echo the continued need for the creation of awareness about the unique situation of IBD patients with regard to clot formation among providers. Further large multicenter studies are needed to further explore these associations.
